# Beryllium – Determination of beryllium and its inorganic compounds in workplace air using atomic absorption spectrometry (GF-AAS): Air Monitoring Method

**DOI:** 10.34865/am744041e11_1or

**Published:** 2026-03-30

**Authors:** Katrin Pitzke, Uta Lewin-Kretzschmar, Ralph Hebisch, Andrea Hartwig, MAK Commission

**Affiliations:** 1 Institute for Occupational Safety and Health of the DGUV (IFA), German Social Accident Insurance (DGUV) Alte Heerstraße 111 53757 Sankt Augustin Germany; 2 German Social Accident Insurance, Institution for the raw materials and chemical industry, Prevention - Department of Hazardous Substances, Biological Agents and Analytical Chemistry Kurfürsten-Anlage 62 69115 Heidelberg Germany; 3 Federal Institute for Occupational Safety and Health (BAuA) Friedrich-Henkel-Weg 1–25 44149 Dortmund Germany; 4 Institute of Applied Biosciences, Department of Food Chemistry and Toxicology, Karlsruhe Institute of Technology (KIT) Adenauerring 20a, Building 50.41 76131 Karlsruhe Germany; 5 Permanent Senate Commission for the Investigation of Health Hazards of Chemical Compounds in the Work Area, Deutsche Forschungsgemeinschaft, Kennedyallee 40, 53175 Bonn, Germany. Further information: Permanent Senate Commission for the Investigation of Health Hazards of Chemical Compounds in the Work Area | DFG

**Keywords:** beryllium, air analyses, analytical method, workplace measurement, hazardous substance, graphite furnace atomic absorption spectrometry, GF-AAS, membrane filter, acid digestion

## Abstract

This analytical method is a validated measurement procedure for the determination of the respirable particle fraction of beryllium [7440-41-7] and its inorganic compounds in workplace air after personal or stationary sampling. Sampling is performed by drawing a defined volume of air through a nitrocellulose membrane filter using a suitable flow-regulated pump. After acid digestion, beryllium and its inorganic compounds retained on the filter are analysed using graphite furnace atomic absorption spectrometry. The relative limit of quantification (LOQ) is 0.0013 µg Be/m^3^ for an air sample volume of 1.2 m^3^. The mean recoveries for beryllium metal and for beryllium from beryllium oxide are 100.1% and 97.1%, respectively. The concentration-dependent expanded measurement uncertainty is 17% to 19% for the respirable und 14% to 16% for the inhalable particle fraction. This analytical method has been accredited by the accident insurance companies for the detection of substances in workplace air that are carcinogenic, mutagenic or toxic to reproduction. This method has been tested and recommended for the determination of beryllium and its inorganic compounds in work areas by the German Social Accident Insurance (DGUV).

Joint Publication of the Analytical Subcommittee of the Chemistry Board of Experts of the Expert Committee Raw Materials and Chemical Industry of the German Social Accident Insurance and the working group “Air Analyses” of the Permanent Senate Commission of the Deutsche Forschungsgemeinschaft for the Investigation of Health Hazards of Chemical Compounds in the Work Area. Based on a German version published by the German Social Accident Insurance in DGUV Information 213-513 Method 03, issued: April 2025.

Please direct correspondence to Berufsgenossenschaft Rohstoffe und chemische Industrie, Prävention, P.O. Box 101480, 69004 Heidelberg, Germany; analytik@bgrci.de

**Table:** 

Name	CAS No.	Molar mass [g/mol]	Symbol	Substance-specific limit values[µg Be/m^3^]^a)^
Beryllium and its inorganic compounds	7440-41-7	9.01	Be	OELV 0.14 (I) EF 1 (I), 0.06 (R) EF 1 (I) (AGS 2025)

OELV: occupational exposure limit value; I: inhalable fraction; R: respirable fraction; EF: excursion factor (peak limitation category)

^a)^ The concentration refers to the metal content of the respective compound.

## 1 Summary

This method involves the use of personal or stationary sampling procedures to determine the mean concentrations of beryllium and its inorganic compounds in the work area over the sampling period. According to Technical Rule (TRGS) 402, the range validated for the procedure is sufficient for determining the respirable fraction of beryllium and its compounds (AGS 2023). The suitability of the method for making determinations in the concentration range of the inhalable fraction must be assessed.

**Table:** 

**Measurement principle:**	A pump is used to draw a defined volume of air through a membrane filter. Following acid digestion, the fraction deposited on the filter is analysed for beryllium by graphite furnace atomic absorption spectrometry (GF-AAS).	
**Limit of quantification:**	Absolute:	0.0016 µg of beryllium per sample carrier
	Relative:	0.0013 µg Be/m^3^ for an air sample volume of 1.2 m^3^ (a sampling period of 2 hours at 10 l/min, a measurement solution volume of 20 ml, a dilution factor of 4 and an injection volume of 20 µl and 5 µl matrix modifier)
**Measurement range:**	Validated: 0.0013–0.12 µg/m³, based on an air sample volume of 1.2 m³	
**Range of the analytical procedure:**	Analytical: 0.05–0.5 µg/l	
**Selectivity:**	The key factors influencing the selectivity of the method are the absorption wavelength, the absence of spectral interference and the minimisation of non-spectral interference.	
**Advantages:**	Measurements with high sensitivity can be taken by personal sampling; suitable for short-term measurements	
**Disadvantages:**	Does not show concentration peaks, requires specialised equipment, time-consuming	
**Apparatus:**	Sampling devices:	
	Pump and sampling head, filter holder with membrane filter and supporting sieve	
	Flow meter	
	Digestion apparatus	
	Atomic absorption spectrometer with graphite furnace (graphite tube with L’vov platform, pyrocoated) and Zeeman background correction	

## 2 Equipment and chemicals

The materials, consumables and chemicals listed in this chapter were used to determine the characteristics of the method. Substitutions may be made if they are of the same quality and purity, e.g. perfluoroalkoxy copolymer (PFA) instead of quartz glass. Consumables and chemicals, etc., must be checked for blank values and sources of interference before use. If these are present, adjustments must be made.

### 2.1 Equipment

For sampling:

- Pump for personal and stationary sampling with a nominal flow rate of 10 l/min (e.g. type SG10-2, from GSA Messgerätebau GmbH, 40880 Ratingen, Germany)
- GSP10 sampling head (e.g. from GSA Messgerätebau GmbH, 40880 Ratingen, Germany)
- FSP10 sampling head with cyclone separator (e.g. from GSA Messgerätebau GmbH, 40880 Ratingen, Germany)
- PGP filter cassette made of plastic, supplied with filter covers 37 mm in diameter (e.g. from GSA Messgerätebau GmbH, 40880 Ratingen, Germany)
- Supporting sieve, 37 mm (e.g. from Metaq GmbH, 42115 Wuppertal, Germany)
- Membrane filter, diameter 37 mm, pore size 8.0 µm, nitrocellulose, preferably with a certified metal content (e.g. from Sartorius AG, 37075 Göttingen, Germany)
- Volumetric flow meter (e.g. TSI model 4140, from TSI GmbH, 52068 Aachen, Germany)

For sample preparation:

- Heating block thermostat made of metal or graphite with time/temperature control, operating range up to 200 °C (e.g. from Gebr. Liebisch GmbH & Co., 33649 Bielefeld, Germany)
- Graduated digestion vessels with air cooler made of quartz glass (diameter 19 mm, maximum volume 25 ml) with ground-glass joints (ST 19/26), acid-proof 0.2-ml graduations from 15 to 25 ml (e.g. from VWR International GmbH, 64295 Darmstadt, Germany)
- Graduated digestion vessels with air cooler made of quartz glass (diameter 23 mm, maximum volume 50 ml) with ground-glass joints (ST 19/26), acid-proof 0.5-ml graduations from 35 to 50 ml (e.g. from VWR International GmbH, 64295 Darmstadt, Germany)
- Glass rods (diameter about 4 mm) made of quartz glass fitted with replaceable endpieces of polytetrafluoroethylene (PTFE) tubing (e.g. from VWR International GmbH, 64295 Darmstadt, Germany)
- Polyethylene stoppers for the digestion vessels (ST 19/26) (e.g. from Pöppelmann GmbH & Co. KG, 49378 Lohne, Germany)
- 5-litre bottle made of PFA with PTFE dispenser for rinsing the air cooler or preparing dilutions (e.g. Optifix HF Dispenser 30 ml from Poulten & Graf GmbH (Fortuna), 97877 Wertheim, Germany)
- 1-litre container made of PFA for diluting acids
- Measuring cylinders made of PFA, 1000 ml, 500 ml, 100 ml, 50 ml (e.g. from VITLAB GmbH, 63762 Großostheim, Germany)
- 2.5-litre quartz bottle with lateral filler neck (ST 29/32), sealable with air-permeable PTFE ground-glass stopper and acid-resistant bottle top dispenser made of PTFE, custom-made (e.g. from VWR International GmbH, 64295 Darmstadt, Germany). Used to dispense freshly prepared standard acid mixtures into digestion vessels
- Ceramic tweezers for transferring the membrane filters to the digestion vessels (e.g. from PLANO GmbH, 35578 Wetzlar, Germany)

For the analytical determination:

- Atomic absorption spectrometer with graphite furnace (GF-AAS), preferably with Zeeman background correction and autosampler; graphite tube with L'vov platform, pyrocoated, and beryllium hollow cathode lamp
- Volumetric flasks made of PFA for standard and calibration solutions with screw caps and ring mark, e.g. 1000 ml, 500 ml, 100 ml, 50 ml, 10 ml (e.g. from VITLAB GmbH, 63762 Großostheim, Germany)
- Reusable PTFE vials, volume 5 ml, for autosamplers (e.g. from Analytik Jena GmbH, 07745 Jena, Germany)
- Disposable polystyrene vials, volume about 1.5 ml for autosamplers (e.g. from Greiner Bio-One, 72636 Frickenhausen, Germany)
- Piston pipettes to cover a volume range from 2 µl to 10 ml, air displacement: for aqueous solutions and suspensions with a density and viscosity similar to water (e.g. Socorex Acura 825 (2 µl to 1000 µl), Socorex Acura 835 (500 µl to 5000 µl and 1000 µl to 10 000 µl), from Socorex Isba S.A., Ecublens, Switzerland)
- Containers, e.g. capacity 2 litres, made of PFA, to store the dilution solution
- Microbalance, readability at least 0.01 mg (e.g. AT 460 DeltaRange, from Mettler-Toledo GmbH, 35396 Gießen, Germany)
- Ultrapure water system with reverse osmosis unit and water purification system for the preparation of ultrapure water (specific resistance ≥ 18.2 MΩ × cm at 25 °C), for the reduction of the overall metal content, in particular for the production of water that is low in boron and alkalis (e.g. GenPure with X-CAD, from Wilhelm Werner GmbH, 51381 Leverkusen, Germany)

### 2.2 Chemicals

- Ultrapure water (specific resistance ≥ 18.2 MΩ × cm at 25 °C)
- Hydrochloric acid, 30%, batch certification of metal concentration by the manufacturer (e.g. Suprapur, from Merck KGaA, 64293 Darmstadt, Germany)
- Nitric acid, 65%, batch certification of metal concentration by the manufacturer (e.g. Suprapur, from Merck KGaA, 64293 Darmstadt, Germany)
- Nitric acid, 67–69%, used to prepare the dilution solution, batch certification of metal concentration by the manufacturer (e.g. INSTRA-ANALYZED Plus for the trace analysis of metals, J.T. Baker, from VWR International GmbH, 64295 Darmstadt, Germany)
- Beryllium plasma standard solution, 1000 µg/ml, Be_4_O(C_2_H_3_O_2_)_6_ in 5% HNO_3_ (e.g. Alfa Aesar Specpure, traceable to NIST SRMs, from Thermo Fisher (Kandel) GmbH, 76185 Karlsruhe, Germany, Order No. 13848)
- Beryllium oxide, 99.95% (metals basis) (e.g. Alfa Aesar, traceable to NIST SRMs, from Thermo Fisher (Kandel) GmbH, 76185 Karlsruhe, Germany, Order No. 35480)
- Beryllium, 99.8% (metals basis), vacuum-packed, beryllium foil, 0.25 mm (0.01 in) thick, equivalent to about 0.29 g/25 × 25 mm (e.g. Alfa Aesar, from Thermo Fisher (Kandel) GmbH, 76185 Karlsruhe, Germany, Order No. 41642)
- Magnesium matrix modifier for GF-AAS, 10.0 ± 0.2 g/l, Mg(NO_3_)_2_ · 6 H_2_O in 17% HNO₃ (e.g. from Merck KGaA, 64293 Darmstadt, Germany, Order No. 1.05813), serves as a Mg stock solution
- Palladium matrix modifier for GF-AAS, 10.0 ± 0.2 g/l, Pd(NO_3_)_2_ in about 15% HNO₃ (e.g. from Merck KGaA, 64293 Darmstadt, Germany, Order No. 1.07289), serves as a Pd stock solution
- Ag, Al, As, Ba, Be, Bi, Ca, Cd, Co, Cr, Cs, Cu, Fe, Ga, Hg, In, K, Li, Mg, Mn, Na, Ni, Pb, Rb, Se, Sr, Tl, U, V and Zn multi-element calibration standard 3 (MES3), 10 µg/ml in 5% HNO_3_, (e.g. from PerkinElmer LAS GmbH, 63110 Rodgau, Germany, Order No.: N9301720)
- Al, Ag, As, B, Ba, Be, Bi, Ca, Cd, Cs, Co, Cr, Cu, Fe, In, K, Li, Mg, Mn, Mo, Na, Ni, Nb, Pb, Rb, Sb, Se, Sr, Ti, Tl, V, U and Zn multi-element quality control standard (QC33), 100 mg/l, in 3% HNO_3_ (e.g. ARISTAR, traceable to NIST SRMs, from VWR International GmbH, 64295 Darmstadt, Germany, Order No. 84791.180)
- Argon 5.0 (purity at least 99.999%)

### 2.3 Solutions

The following solutions are prepared using the chemicals listed in Section 2.2. The preparation of further solutions is described in the relevant sections.

**Acid digestion mixture:** solution made of 65% nitric acid and 25% hydrochloric acid 2 : 1 (v : v) (Pitzke et al. 2018, 2020)

The 25% hydrochloric acid solution is prepared by placing 185 ml ultrapure water into a 1-l PFA vessel and mixing it with 815 ml HCl (30%).

1400 ml 65% nitric acid is placed into a 2.5-l quartz bottle and 700 ml of the pre-mixed hydrochloric acid (25%) is added.

**Dilution and blank solution:** solution of about 0.7% HNO_3_ in water (used for the dilution and stabilisation of samples and standards)

About 1.5 l ultrapure water is placed into a 2-l PFA container, 20 ml 67–69% nitric acid is added. The container is filled to 2 l with ultrapure water and then shaken.

**Rinsing solution for GF-AAS**: solution of 3.3% HNO_3_ in water

About 900 ml ultrapure water is placed into a 1000-ml volumetric flask, 5 ml 65% nitric acid is added. The flask is filled to 1000 ml with ultrapure water and then shaken.

**Modifier solution:** solution of 0.1 g Mg/l and 1 g Pd/l in about 1.6% aqueous HNO_3_

About 5 ml ultrapure water is placed into a 10-ml volumetric flask, 0.1 ml Mg stock solution and 1 ml Pd stock solution are added. The flask is filled to 10 ml with ultrapure water and then shaken.

**Quality control solution**: solution of 0.2 µg Be/l and 0.1 µg Be/l in about 0.7% aqueous HNO_3_

The quality control samples are prepared using the multi-element quality control standard (QC33) described in Section 2.2. The preparation of the quality control samples is described in detail in Section 5.2.

**Spiking solutions for precision analysis:** solution of 3.0 mg Be/l, 1.5 mg Be/l and 0.18 mg Be/l in about 0.7% aqueous HNO_3_

The spiking solutions are prepared with the beryllium plasma standard solution described in Section 2.2. The preparation of the spiking solutions is described in detail in Section 6.1.

Acid-stabilised solutions containing metal in concentrations above 1 mg/l can be stored for at least 4 weeks. Acid-stabilised solutions containing metal in concentrations from 0.1 mg/l to 1 mg/l can be stored for at least 2 weeks. Acid-stabilised solutions containing metal in concentrations below 0.1 mg/l should not be used for more than 2 days. These are recommenda­tions.

Calibration and control samples must be prepared fresh every working day.

## 3 Sampling

Before samples are taken, the lot of filters used for testing must be checked for metals (filter lot blank data) to determine whether the filters fulfil the minimum requirements to ensure the effectiveness of the measurement procedures (DIN 2021).

A membrane filter (Ø 37 mm) and a supporting sieve are placed into a filter capsule. The capsule is then inserted into a sampling head for the sampling of the inhalable particle fraction (GSP10) or into a head for sampling the respirable particle fraction (FSP10). The sampling head is connected to the pump. The volumetric flow rate is set to 10 l/min. The definition of inhalable or respirable dust is fulfilled at this volumetric flow rate (DIN 1993). This corresponds to an air sample volume of 1.2 m³ over a sampling period of 2 hours.

Samples are collected using stationary or personal sampling procedures. The inlet of the sampling head must remain unobstructed during sampling.

The flow rate must be checked for constancy after sampling. If the deviation from the adjusted flow rate is > ± 5%, further samples should be taken (see DGUV Information 213-500 “General Part”, Section 3 (DGUV 2015)).

The loaded membrane filter is removed from the sampling system, the capsule is sealed with a cap and then transported to a laboratory for analysis with as little jarring as possible.

## 4 Analytical determination

### 4.1 Sample preparation

Ceramic tweezers are used to carefully fold the loaded membrane filter and transfer it to a 25-ml digestion vessel. The filter is pressed down to the bottom of the vessel with a glass rod. 10 ml of the standard acid digestion mixture is added to the filter. An air cooler is attached to the vessel, which is then placed in a heating block thermostat and heated for two hours to boiling (block temperature: about 135 °C). After cooling down to a temperature of about 50 °C, 10 ml ultrapure water is carefully added through the air cooler to both rinse the cooler and dilute the solution, which will be slightly viscous in some cases. The solution is again heated to boiling. After cooling, the air cooler and the glass rod are removed, the digestion vessel is plugged with a polyethylene stopper and the volume of the sample solution is read off.

Before performing the analysis, the digestion solution is diluted to a ratio of 1 : 4 (v : v) by adding dilution solution (see Section 2.3) using the autosampler of the GF-AAS. The sample is then analysed. Each sample is analysed in duplicate and the mean value is used to calculate the results.

A blank value is determined for each series of samples. For this purpose, all sample preparation and analysis steps are performed with at least two clean, unexposed filters from the same lot. The blank value that is obtained must lie within three standard deviations of the mean blank value determined during the development of the method as described in Section 6.3. If this is not the case, a new mean blank value must be determined.

### 4.2 Operating conditions

The characteristics of the method described in Section 6 were obtained under the following operating conditions:

**Table:** 

**Apparatus:**	Atomic absorption spectrometer with Zeeman graphite furnace system “AAS PinAACle 900Z” from PerkinElmer LAS with “Furnace Autosampler AS900”, integrated recirculating chiller, Advanced Platform THGA, graphite tube with caps, pyrocoated, beryllium hollow cathode lamp, warm-up period of at least 30 minutes
**Absorption wavelength: **	234.86 nm
**Slit width: **	0.7 nm
**Lamp current:**	7 mA
**Background correction:**	Longitudinal Zeeman background correction with alternating current
**Measurement solution:**	An aliquot of the digestion solution (see Section 4.1) is diluted with the dilution solution described in Section 2.3 to a ratio of at least 1 : 4 (v : v). If the results lie outside of the linear range of the calibration function, further dilutions, e.g. 1 : 40 and 1 : 400 (v : v), must be prepared.
**Injection volume:**	20 µl measurement solution and 5 µl matrix modifier, injection by autosampler. After transferring the measurement solution and modifier to the graphite tube, the injection loop must always be rinsed by adding 5 µl dilution solution by autosampler.

All solutions, particularly the calibration solutions and the quality control samples (see Sections 5.1 and 5.2), must be prepared fresh every working day. The GF-AAS temperature/time programme is shown in Table 1.

**Tab. 1:** Temperature/time programme

**Stage**	**Explanation**	**Heating rate** **[°C/s]**	**Furnace temperature** **[°C]**	**Dwell time** **[s]**	**Argon purge gas** **[ml/min]**
1	injection/drying	1	120	15	250
2	drying	15	140	60	250
3	pyrolysis	10	1000	20	250
4	atomisation/measurement	0	2250	3	0
5	cleaning	1	2450	3	250

The prepared sample solutions, blank solutions and filter blank solutions are analysed by GF-AAS. For this purpose, the sample solutions and filter blank solutions are first diluted with the dilution solution described in Section 2.3 in a ratio of 1 : 4 (v : v).

20 µl of each of the test solutions is injected by autosampler into the graphite tube with 5 µl of modifier solution and analysed by AAS under the operating conditions listed above. Each sample is analysed in duplicate and the mean value is used to calculate the results. The AAS system is then cleaned with the rinsing solution described in Section 2.3.

If the concentrations determined for beryllium lie above the calibrated range, the sample solutions are diluted further as described above and analysed again.

Quality control samples are analysed after calibration, after running at most 20 to 25 samples and at the end of a sequence. The samples must fall within the defined limits (± 10% of the nominal value); if not, the calibration must be checked and the sample solutions analysed again. This checks the stability of the analytical equipment and the accuracy of the results.

The baseline is adjusted electronically after each measurement. The baseline is checked regularly, but at least after every 20 to 25 samples, by running the blank solution and setting the signal (area) to zero. Afterwards, the instrument must be completely recalibrated.

## 5 Evaluation

### 5.1 Calibration

The analytical instrument must be calibrated every working day.

To prepare the calibration solutions (see Table 2), a beryllium standard solution of 50 µg/l is first prepared with the 29-element solution standard (10 mg/l, see Section 2.2). For this purpose, 500 µl of the 29-element solution standard is transferred to a 100-ml volumetric flask by pipette, the flask is filled to the mark with dilution solution (see Section 2.2) and then shaken.

A beryllium calibration solution containing beryllium in a concentration of 0.5 µg/l is prepared with the beryllium standard solution (50 µg/l). For this purpose, 1000 µl of the beryllium standard solution (50 µg/l) is pipetted into a 100-ml volumetric flask, the flask is filled to the mark with dilution solution and then shaken.

An autosampler is used to prepare calibration solutions containing beryllium in the concentrations listed in Table 2 from the 0.5 µg/l beryllium calibration solution; the calibration solutions are then measured. The calibration solutions are diluted automatically with the dilution solution (see Section 2.2). The sample injected for calibration has a total volume of 25 µl, including 5 µl of matrix modifier that is added by pipette via the autosampler. Table 2 shows the dosing scheme used to prepare the calibration solutions. The standards are analysed by AAS under the operating conditions described in Section 4.2.

**Tab. 2:** Dilutions used to derive the calibration functions

**Calibration solution**	**Volume of the beryllium calibration solution (0.5 µg Be/l)** **[µl]**	**Volume of the dilution solution** **[µl]**	**Volume of the matrix modifier** **[µl]**	**Concentration** **[µg Be/l]**
0	0	20	5	0
1	2	18	5	0.05
2	4	16	5	0.10
3	6	14	5	0.15
4	8	12	5	0.20
5	10	10	5	0.25
6	12	8	5	0.30
7	14	6	5	0.35
8	16	4	5	0.40
9	18	2	5	0.45
10	20	0	5	0.50

The absorption is determined based on the peaks and plotted against the concentrations. The calibration function is linear under the given conditions.

Higher concentrations of beryllium can be determined in the sample solution by making suitable dilutions. Lines and signals must always be checked carefully because spectral interference may occur.

### 5.2 Quality control

Quality control procedures are performed during an analytical run using commercially available solutions (multi-element quality control standard, see Section 2.2). The aim is to verify the accuracy of the calibration and the stability over time each working day and over the entire measurement period.

The quality control samples are made by first preparing a 200 µg/l multi-element quality control standard stock solution. For this purpose, 100 µl multi-element quality control standard is pipetted into a 50-ml volumetric flask. The flask is filled to the mark with the dilution solution described in Section 2.3 and then shaken. 50 µl and 25 µl, respectively, of the multi-element quality control standard stock solution (200 µg/l) are pipetted into separate 50-ml volumetric flasks, the flasks are filled to the mark with dilution solution and then shaken. The resulting samples are the quality control sample 1 containing 0.2 µg/l beryllium and the quality control sample 2 containing 0.1 µg/l beryllium (see Table 3).

**Tab. 3:** Dilutions used to prepare the quality control samples

**Quality control sample**	**Volume of stock solution** **[µl]**	**Concentration of quality control sample** **[µg Be/l]**
1	50	0.2
2	25	0.1

### 5.3 Calculation of the analytical result

The beryllium concentration in the workplace air is calculated based on the beryllium concentration in the measurement solution, which is calculated using a software programme, and the absorbance values. The data analysis software uses the calculated calibration function for this purpose. The beryllium concentration in the workplace air is calculated from the beryllium concentrations taking the respective dilutions and the air sample volume into account.

Equation 1 is used to calculate the mass concentration of beryllium in the air sample in µg/m³:



(1)

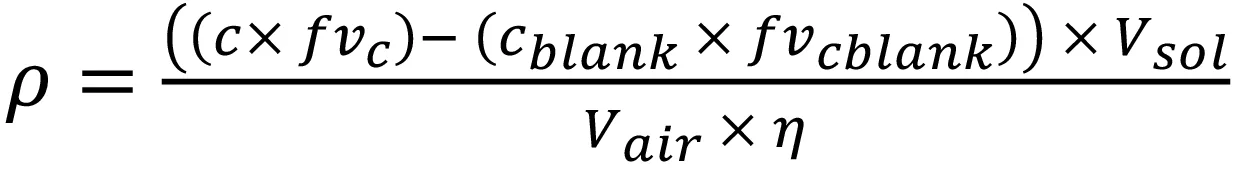

rho equals startFraction open parenthesis open parenthesis c times fv subscript c close parenthesis minus open parenthesis c subscript blank times fv subscript cblank close parenthesis close parenthesis times V subscript sol divided by V subscript air times eta endFraction



where:

**Table:** 

*ρ*	is the mass concentration of beryllium in the air sample in µg/m^3^
*c *	is the concentration of beryllium in the measurement solution in µg/l
*fv_c_*	is the dilution factor of the sample, here 1 : 4
*c_blank_*	is the concentration of beryllium in the blank samples in µg/l
*fv_cblank_*	is the dilution factor, here 1 : 4
*V_sol_*	is the volume of the prepared sample solution in l
*V_air_*	is the air sample volume in m³
*η*	is the recovery

## 6 Reliability of the method

The characteristics of the method were calculated according to DIN EN 482 (DIN 2021), TRGS 402 (AGS 2023), DIN EN ISO 21832 (DIN 2020), ISO 20581 (ISO 2016) and DIN 32645 (DIN 2008).

### 6.1 Precision

The precision of the method was determined by spiking sets of six membrane filters with different masses of beryllium using the spiking solutions described below.

Beryllium spiking solution 1 (3.0 mg/l) was prepared by placing a few millilitres of dilution solution (Section 2.3) into a 100-ml volumetric flask and then adding 300 µl of the beryllium plasma standard solution (1000 mg/l) (see Section 2.2) by pipette. The flask was then filled to the mark with dilution solution. A similar procedure was used to prepare beryllium spiking solution 2 (1.5 mg/l): 150 µl beryllium plasma standard solution (1000 mg/l) was pipetted into a 100-ml volumetric flask and the flask was then filled to the mark with dilution solution. Beryllium spiking solution 3 (0.18 mg/l) was prepared by pipetting 3 ml beryllium spiking solution 1 (3.0 mg/l) into a 50-ml volumetric flask and filling the flask to the mark with dilution solution.

Nitrocellulose filters were spiked with 40 µl beryllium spiking solution 3 (0.18 mg/l) to determine the precision at 0.1 times the occupational exposure limit value (OELV), with 48 µl beryllium spiking solution 2 (1.5 mg/l) for the precision at the OELV and with 48 µl beryllium spiking solution 1 (3.0 mg/l) for the precision at 2 times the OELV.

The filters were dried under the fume hood at room temperature for 24 hours and then underwent digestion and analysis as described in Section 4.1 (see Sections 4.2 and 5).

At an air sample volume of 1200 l, the spiked amounts are equivalent to the air concentrations listed in Table 4.

Prior to analysis by GF-AAS, the digestion solutions were diluted by a factor of 4 with the dilution solution described in Section 2.3. In addition, a complete analytical run was performed with two clean, unexposed filters to obtain blank values.

The results were used to determine the data for precision given in Table 4.

**Tab. 4:** Precision data

**Spiked mass of beryllium** **[µg]**	**Concentration tested^a)^** **[µg/m³]**	**Relative standard deviation** **[%]**
0.0072	0.0060	2.3
0.072	0.060	1.4
0.14	0.12	1.5

^a)^ The concentration was calculated based on a 2-hour sampling period at a volumetric flow rate of 10 l/min.

### 6.2 Recovery

The aerosols that occur in different work areas are of varying chemical composition and have different physical properties; it is therefore not possible to determine recovery data that are generally valid for the entire procedure.

The analytical recovery is defined as 100% according to DIN EN ISO 21832 based on the sample preparation method (see Section 4.1) (valid only for those metals and compounds that are soluble in the described system) (DIN 2020).

Certified beryllium and a certified beryllium compound (see Section 2.2) were used to evaluate the sample preparation method described above with regard to the analytical recovery and the reproducibility of the method. The recovery experiments were performed with beryllium (99.8%, metals basis) and beryllium oxide (99.95%, metals basis).

Concentrations equivalent to 0.1, 1 and 2 times the OELV of beryllium (respirable dust) were included to cover the minimum measurement range. In this experiment, not only the recovery, but also the relative standard deviation for each concentration was calculated as a measure of reproducibility. As the concentrations lie in the ultratrace range, it is difficult to determine the weight of the reference materials reliably. The following procedure was therefore used:

Two samples, Samples A and B, each containing about 10 mg of the substances listed in Table 5, were weighed onto nitrocellulose filters. The filters were transferred to 25-ml digestion vessels and 10 ml of the acid digestion mixture (see Section 2.3) was added. Glass rods were placed into the vessels, and the samples were then heated for 2 hours to boiling at a block temperature of 135 °C. After cooling to about 50 °C, 10 ml of ultrapure water was added to each sample through the air cooler and the samples were then heated again at a temperature of 135 °C for about 30 minutes. The solutions prepared as described did not contain any visible particles. The vessels were filled with ultrapure water to a final volume of 20 ml.

Calculations were performed to determine the beryllium concentrations in the solutions made with the pure substance according to this method. For the beryllium metal, Sample A contained a beryllium concentration of 548.90 mg/l and Sample B a beryllium concentration of 518.96 mg/l. For beryllium oxide, Sample A contained a beryllium concentration of 194.54 mg/l and Sample B a beryllium concentration of 192.74 mg/l. These solutions were diluted until the beryllium concentrations at analysis were 0.0060 µg/m³ (equivalent to 0.1 OELV, 7.2 ng absolute), 0.06 µg/m³ (equivalent to 1 OELV, 72.0 ng absolute) and 0.12 µg/m³ (equivalent to 2 OELV, 144 ng absolute).

Additionally, all steps of the analytical procedure were carried out with at least two clean, unexposed filters (blank filters). The blank value did not need to be corrected for the calculation of the results.

The quantitative analysis resulted in a mean recovery of 100.1% for beryllium in the form of beryllium metal and 97.1% for beryllium obtained from beryllium oxide.

Correction by the recovery value was not required for the analytes described here.

Table 5 shows the mean recovery data for the reference materials mentioned above.

**Tab. 5:** Mean recovery for beryllium with n = 6 determinations

**Test substance**	**Mass of beryllium on the filter^a)^** **[ng]**	**Beryllium concentration^b)^** **[µg/m³]**	**Mean recovery** **[%]**	**Relative standard deviation** **[%]**
Beryllium	7.2	0.0060	96.7	1.0
Beryllium	72	0.060	101.5	1.3
Beryllium	140	0.12	102.2	1.2
Beryllium oxide	7.2	0.0060	95.6	2.2
Beryllium oxide	72	0.060	96.6	1.7
Beryllium oxide	140	0.12	99.2	1.5

^a)^ The masses were not determined directly by weighing, but by reverse calculation after factoring in the dilution steps and the digestion volume.

^b)^ The concentration is obtained for a sampling period of 2 hours at a volumetric flow rate of 10 l/min and a digestion volume of 20 ml.

### 6.3 Limit of quantification

The procedure used to calculate the limit of quantification during method development was similar to the blank value method described in DIN 32645 (DIN 2008). For this purpose, all preparation and analysis steps were carried out with 10 unexposed membrane filters and the results were used to determine the limit of quantification. The mean values, the analyte blank values obtained from the filters, reagents and vessels used in the experiments and the corresponding standard deviation were calculated. The relative limit of quantification was calculated using the value equivalent to ten times the standard deviation of the blank values in the measurement solution, the digestion volume of 20 ml, the dilution factor of 4 and an air sample volume of 1.2 m³.

The value obtained for the relative limit of quantification based on these parameters was 0.0013 µg/m³ beryllium, or 0.0016 µg of beryllium per sample carrier (absolute).

### 6.4 Storage stability

The storage stability of the loaded membrane filters was analysed by spiking the filters with the spiking solutions described in Section 2.3.

The filters were spiked with beryllium and the samples were prepared using the method described in Section 6.1.

All preparation and analysis steps were carried out with 3 filters per concentration on two days a week over a period of four weeks. The recovery remained stable in the range of 0.006 µg/m³ to 0.12 µg/m³ over the investigated time period.

Beryllium concentrations close to the limit of quantification will remain stable if stored for up to 14 days. However, a storage period of 14 days should not be exceeded.

### 6.5 Selectivity

The key factors that influence the selectivity of the method are the choice of wavelength and the absence of spectral interference.

The standard addition procedure should be used in the case of non-spectral interference such as complex matrix effects. Electrodeless discharge lamps (EDL) are recommended for use as a radiation source, because these have a considerably higher radiation flux density than hollow cathode lamps (HCL). Measurements taken with EDL are significantly more sensitive and EDLs have a better signal-to-noise ratio (Doerffel and Eckschlager 1981).

The matrix of the samples under analysis is an important factor in determining the wear on the graphite tube or the graphite platform of the GF-AAS. If the composition of the samples is unknown, this requires longer heating steps at higher temperatures, which shortens the service life of the graphite components considerably. Therefore, the condition of the graphite components must be checked regularly, e.g. based on the recovery of the control samples.

### 6.6 Expanded measurement uncertainty

The expanded measurement uncertainty was determined by estimating all relevant influencing parameters. The two main sources of uncertainty in the measurement results are uncertainties in the sampling procedure and uncertainties in the analytical procedure.

The uncertainties arising from the method were estimated by determining the uncertainties associated with the air sample volume and the sampling effectiveness for respirable and inhalable dusts (DIN 2020).

Uncertainties arising from the analysis may occur at any point of the analytical procedure including digestion, dilution, calibration, recovery and precision. The “QMSys GUM PRO” ([Bibr ref_CSPX7P4Y]) software was used to determine the analytical uncertainties for this method that vary depending on the concentration; these are listed in Table 6. The software was developed based on the EURACHEM/CITAC Guide “Quantifying uncertainty in analytical measurements” (Ellison and Williams 2012) and the ISO publication “Guide to the expression of uncertainty in measurement (GUM)” (ISO 2008) and fulfils the requirements of the German standard DIN EN ISO/IEC 17025 (DIN 2018).

The combined uncertainties for the entire method were calculated by combining the contributions from all sources of uncertainty. The percentages listed in Table 6 for the expanded measurement uncertainty (*U*) of the entire method for the tested concentrations of beryllium at a volumetric flow rate of 10 l/min and a sampling period of 2 hours were obtained by multiplying these values with the expansion factor k = 2.

**Tab. 6:** Expanded measurement uncertainty *U* with n = 6 determinations

**Metal**	**Fraction**	**Concentration tested** **[µg/m³]**	**Expanded measurement uncertainty** **[%]**
Beryllium	respirable particle fraction	0.0060	18.6
		0.060	17.1
		0.12	16.9
Beryllium	inhalable particle fraction	0.0060	15.4
		0.060	14.6
		0.12	13.5

### 6.7 Comparison of open hot-block digestion with microwave-assisted pressure digestion

The recovery experiments carried out to validate the method were also used to compare the open hot-block digestion method described here with microwave-assisted pressure digestion. The latter is included as an alternative digestion method in the MAK Collection published by the German Research Foundation (DFG) and in the IFA Folder of the German Institute of Occupational Safety and Health (IFA-Arbeitsmappe) (Pitzke et al. 2018, 2020). The samples for microwave-assisted pressure digestion were prepared by weighing about 10 mg of the substances listed in Section 6.2, Table 5 onto nitrocellulose filters. The filters were transferred to PTFE vessels and 10 ml nitric acid (65%, see Section 2.2) was added. The vessels were sealed with PTFE caps and underwent digestion for 65 minutes at 250 °C and 140 bar. After cooling, the digested samples were transferred to 25-ml digestion vessels, ultrapure water was added to obtain a final volume of 20 ml and the vessels were then shaken. The solutions prepared as described did not contain any visible particles. Calculations were performed to determine the beryllium concentrations in the solutions made with the pure substance. For the beryllium metal, Sample A contained a beryllium concentration of 518.96 mg/l and Sample B a beryllium concentration of 568.86 mg/l. For beryllium oxide, Sample A contained a beryllium concentration of 180.13 mg/l and Sample B a beryllium concentration of 183.73 mg/l. The digestion solutions were diluted using the same procedure as for the digestion samples that underwent open hot-block digestion and then analysed. The mean recovery for beryllium in the form of beryllium metal was 99.1% and 95.9% for beryllium obtained from beryllium oxide.

The recovery data obtained by open hot-block digestion and by microwave-assisted pressure digestion are comparable; however, microwave-assisted pressure digestion had a higher coefficient of variation (> 5%). No other characteristics were determined for microwave-assisted pressure digestion.
